# Cross-Modality Person Re-Identification Based on Heterogeneous Center Loss and Non-Local Features

**DOI:** 10.3390/e23070919

**Published:** 2021-07-20

**Authors:** Chengmei Han, Peng Pan, Aihua Zheng, Jin Tang

**Affiliations:** 1 Anhui Provincial Key Laboratory of Multimodal Cognitive Computation, School of Computer Science and Technology, Anhui University, Hefei 230601, China; cmhan@hfnu.edu.cn (C.H.); e20301149@stu.ahu.edu.cn (P.P.); ahzheng214@foxmail.com (A.Z.); 2 School of Computer Science and Technology, Hefei Normal University, Hefei 230601, China

**Keywords:** cross-modality, person re-identification, heterogeneous center loss, non-local

## Abstract

Cross-modality person re-identification is the study of images of people matching under different modalities (RGB modality, IR modality). Given one RGB image of a pedestrian collected under visible light in the daytime, cross-modality person re-identification aims to determine whether the same pedestrian appears in infrared images (IR images) collected by infrared cameras at night, and vice versa. Cross-modality person re-identification can solve the task of pedestrian recognition in low light or at night. This paper aims to improve the degree of similarity for the same pedestrian in two modalities by improving the feature expression ability of the network and designing appropriate loss functions. To implement our approach, we introduce a deep neural network structure combining heterogeneous center loss (HC loss) and a non-local mechanism. On the one hand, this can heighten the performance of feature representation of the feature learning module, and, on the other hand, it can improve the similarity of cross-modality within the class. Experimental data show that the network achieves excellent performance on SYSU-MM01 datasets.

## 1. Introduction

### 1.1. Definition of Person Re-Identification

Person re-identification technology (Re-ID) uses the whole body image of pedestrians for identity recognition, which can extend the space-time continuity of the continuous tracking of pedestrians under cameras. The research goal of person re-identification technology is to determine whether a pedestrian appears under multiple non-overlapping independent surveillance cameras [1,2,3]. In practical applications, the technology is facing great challenges due to the influence of diverse factors such as light changes, different perspectives, human postures, and so on.

Early person re-identification technology consisted of studies under visible light modality (single modality). There are two key points in the research: one is feature extraction, the other is metric learning. Feature extraction means extracting the image features of the target pedestrian and candidate pedestrian images [1,2,3,4,5]. The distance between the target pedestrian and candidate pedestrian images features is calculated by metric learning, and then the similarity values between them are calculated. Most of the traditional feature extraction algorithms use artificial feature extraction methods [1,2,6,7,8,9,10]. Since the application of deep learning to computer vision, the recognition accuracy of single-modality pedestrian re-identification [11,12,13,14,15,16,17] has reached new stage, which has even exceeded the ability of human re-identification.

### 1.2. Cross-Modality Person Re-Identification

Due to the increasing demand for person re-identification technology in practical application scenarios, person re-identification of a single modality is also facing new problems. Single-modality person re-identification [11,12,13,15,18,19] mainly uses the pedestrian images collected by visible light cameras for recognition. However, under the condition of insufficient light or night, the information of the images collected by visible light cameras is seriously missing, which affects the effect of person recognition. With the reduction in hardware costs and the progress of webcam technology, advanced monitoring instruments have been equipped with dual monitoring modes under visible light and dark conditions. The visible light monitoring mode is used when the lighting conditions are good in the daytime, and the infrared mode is automatically switched to monitoring when the light is weak at night. Cross-modality person re-identification [5,20,21,22,23,24,25] is a technology to study whether the target pedestrian appears in the cross-modality surveillance cameras. At present, the commonly used cross-modality cameras are visible-infrared cameras and visible-thermal imaging cameras. Cross-modality person re-recognition technology mainly studies the matching technology of pedestrian images in different modalities. For example, given an RGB image of a pedestrian collected under visible light, the technology can judge whether the pedestrian appears in the infrared images (IR images) collected at night, and vice versa.

### 1.3. The Progress and Challenges of Cross-Modality Person Re-Identification

**Figure 1:**
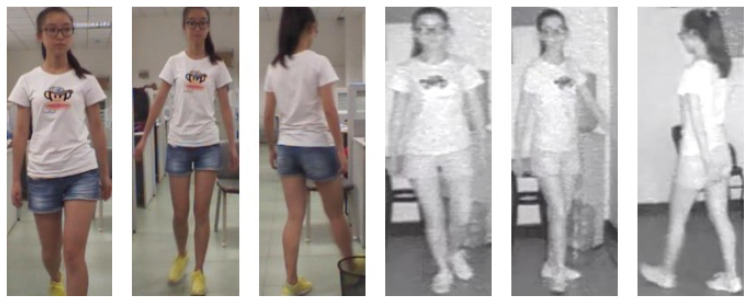
Cross-modality differences of the same pedestrian. The three images on the left and the three images on the right are RGB modality images and IR modality images of the same person, respectively.

The difficulties of cross-modality person re-identification technology consist of studying the common characteristics of the same pedestrian in different modalities and reducing the modal difference of the same pedestrian in the two modalities. The main methods of cross-modality pedestrian Re-ID [20,21,23,26,27] are to design a more optimized convolutional neural network for feature extraction and design a suitable loss function. However, the IR images in cross-modality person recognition lack a lot of color information, which leads to great differences in the characteristics of the same pedestrian in different modalities, as shown in Figure 1. At present, the mainstream process of cross-modality person re-identification is to extract the features of RGB images and infrared images by using a deep neural network of a two-way structure, then using the shared parameter network to learn the features of the two modalities, and finally, realizing the re-identification. However, in designing the loss functions, most methods consider expanding the differences among different pedestrian categories. However, it is insufficient to learn the similarity of the same pedestrian in different modalities.

At present, the methods of RGB image person re-identification have achieved remarkable performance. The deep neural networks, which have made great progress for single-modality person re-identification, have also boosted cross-modality person re-identification. However, this new task still faces the problem of intra-class modality difference, as shown in Figure 1. Therefore, cross-modality research not only focuses on improving the feature extraction ability of the deep network but also considers how to design appropriate loss functions to improve the feature similarity value of the same pedestrian in the different modalities. Zhu et al. [23] uses heterogeneous loss function to improve the similarity of the same person under different modalities and achieves superior performance. However, they do not consider the contribution of global features to each location. To further improve the network’s contribution to cross-modality person Re-ID, inspired by [23,25,28], we propose to improve the feature expression ability and intra-class cross-modality similarity from two aspects, namely improving the structure of a deep network and designing a loss function. In the following description, we design a dual-channel deep neural network framework that combines a heterogeneous center loss function and non-local module. The main contribution of this documentation is based on a two-way network (RGB branch and IR branch) in order to extract the features of visible-light images and IR images separately. We propose improving the contribution of global features to each location. Specifically, inspired by [25,28], we propose to add a non-local attention module to the RGB branch and IR branch to improve the contribution of global features to each local location. The structure of the two network channels is the same, but the parameters are not shared, and the network structure has scalability. To improve the feature similarity of intra-class cross-modality images, inspired by HC loss [23], the heterogeneous center loss function is used to shorten the distance between different modality feature centers in the class. Therefore, this paper combines cross-entropy loss (CE loss) and the heterogeneous center loss (HC loss) function to supervise training jointly. Beyond this, because different parts of a human body have their inherent properties, the idea of the block is introduced into the network to obtain the characteristic expression of discriminating force between different modalities. The performance of the proposed method on the SYSU-MM01 [26] dataset demonstrates the effectiveness.

## 2. Related Work

### 2.1. Visible Light Modality Person Re-ID

In visible light modality person re-identification, there are three main directions in the aspect of pedestrian feature expression: global feature extraction, local feature extraction, and feature extraction with attention mechanism. Global feature extraction: in the task of image classification, global feature extraction has been treated as the primary choice to obtain the features of images [4,29,30,31]. Global feature extraction consists of learning the global feature vector representation [32] of person images through the deep neural network. The IDE model [32] treats the recognition task of a pedestrian image as a classification task and uses global feature extraction for person Re-ID. Many works [11,33] prove the validity of the IDE. Local feature learning: due to the distinguishable features of various parts of the human body, the PCB [11] proposes that the horizontal direction of the pedestrian feature vectors are divided into several equal parts by using the idea of a horizontal block, and then the local feature vectors of pedestrians are learned through the network. Finally, each local feature is connected into a whole for person re-identification. On the principle of PCB [11], some works [18,34] improve the algorithm and increase the recognition accuracy of the person Re-ID task. Feature extraction with attention mechanism: the conventional deep convolution neural network uses a convolution kernel with a convolution kernel of 3 or 5 to extract the local features of the image, ignoring the contribution of the global characteristics of the image to person image recognition. Wang et al. [28] use non-local operations to improve the contribution of image global features by associating the information of any two locations. The idea based on the non-local mechanism shows promising performance in person re-identification [14,35].

### 2.2. Cross-Modality Person Re-ID

Cross-modality person re-identification [22,25,26,27,36,37,38,39] consists of solving the problem of person Re-ID under low light or night conditions. Wu et al. [26] for the first time propose the deep zero-padding method for this task. Meanwhile, one-stream, two-stream, and asymmetric FC network structures are proposed for RGB-IR-based person Re-ID [26,40]. At the same time, they contribute a standard benchmark SYSU-MM01 [26] for cross-modality person Re-ID, and supporting evaluation criteria were released. Ye et al. [20] propose a two-flow network using the top loss of the bi-directional central constraint to reduce the modal difference. For a query image, the distance between it and images of the same type should be less than the distance between it and images of different types [20]. Inspired by the central loss in face recognition, Zhu et al. [23] suggest using the feature center distance of the heterogeneous image instead of the feature distance. Zhu et al. [23] propose using the heterogeneous center loss function to reduce the center distance of the feature distribution of the same pedestrian in different modalities: that is, to improve the feature similarity between different modalities within the class. Hao et al. [41] apply the sphere softmax loss (usually used in face recognition) to cross-modality person Re-ID, and Hao et al. [41] propose a hypersphere manifold embedding network (HSMEnet). In cross-modality person Re-ID, cmGan [42,43] also attracts more attention and makes good progress.

## 3. Methods

### 3.1. Overview of a Deep Neural Network Framework Combining Heterogeneous Center Loss and Non-Local Modules

**Figure 2:**
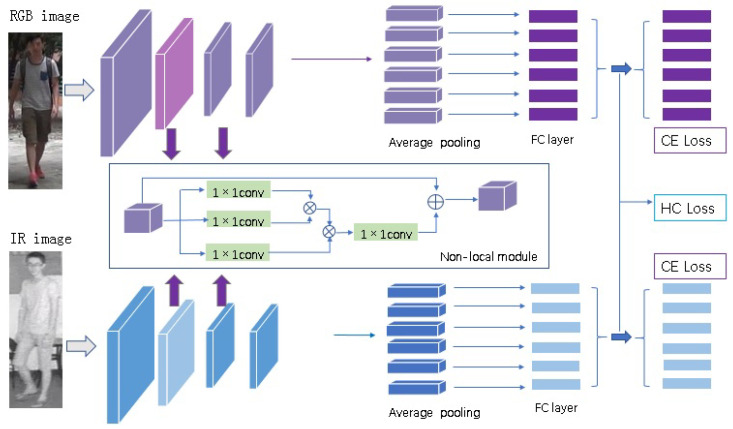
The framework of deep neural network combining heterogeneous center loss and non-local mechanism. It uses a dual-channel structure for feature extraction of RGB images and IR images. We add non-local module to the second layer and the third layer to enhance the contribution of the global features of the image to the location features. After the fourth layer, the feature maps are divided into six blocks in the horizontal direction. CE loss and HC loss are used for training supervision jointly.

The framework of deep neural network combining heterogeneous center loss and non-local module is shown in Figure 2. The framework uses a dual-channel structure for feature extraction of the RGB images channel and the IR images channel. Resnet50 is used as the backbone for each channel. Compared with Resnet50, there are three main differences. One is to remove the down sampling operation in the fourth layer before the average pooling operation. In this way, a larger feature map can be obtained and more feature information can be obtained. Another more important difference is that inspired by [25,28], we add non-local module (which will be introduced in Section 3.2) to the second layer and the third layer to enhance the contribution of the global features of the image to the location features. Thirdly, after the fourth layer, the feature map is divided into *P* blocks (P=6) in the horizontal direction by using the idea of PCB [11]. The dimension of each block is reduced by using 1×1 convolution; then, L2-norm layer operation and FC layer operation are performed. Finally, HC loss (which will be introduced in Section 3.3) and CE loss are calculated.

### 3.2. Non-Local Module

In the application of computer vision, the common convolution operation is to extract local features. The non-local operation [28] is to calculate the relationship between the pixels in two different positions on the image. That is to say, it is used to capture long-range dependencies. When calculating the response of a pixel, non-local operations take into account the contribution of features in all positions. Let the receptive field of convolution be larger, not limited to a local area. The general expression of non-local [28] is as follows:

(1) $$\begin{array}{c} y_{i}=\frac{1}{C\left(x\right)}\sum_{\forall j}^{}f\left(x_{i},x_{j}\right)g\left(x_{j}\right), \end{array}$$

where *x* represents the input feature map, *i* represents the response of the current position, and *j* represents the response of each position on the feature map. Given the corresponding *i* in a certain position, the global response is obtained by weighted summation of the response *j* in each position. $f\left(x_{i},x_{j}\right)$ is used to calculate the similarity between *i* and all possible associated locations *j*. We use $g\left(x_{i}\right)$ to represent the feature representation of the feature map at the *j* position. $C\left(x\right)$ is the standardized factor. Finally, we obtain *y* with the same size as *x*. Non-local is used in the shallow layer of CNN and before the layer of FC. FC uses the learned weights to calculate the mapping from input to output. In the layer of FC, the relationship between position *i* and position *j* will not be reflected in the output, and the position correlation is lost to a certain extent. By combining the information of non-local and local, we can make up for this loss and extract more abundant features.

### 3.3. Metric Learning Module

In cross-modality pedestrian images, RGB images and IR images are called heterogeneous images due to different imaging principles. The contour information and texture information of the same individual will remain consistent in different modalities, but the color information will change greatly in different modalities. The traditional cross-entropy loss is used to push the distance between different types of pedestrian images. However, it cannot help to shorten the feature distance of the same category of pedestrians under different modalities. Inspired by the central loss function used in face recognition [44], Zhu et al. [23] propose a heterogeneous center loss function. By penalizing the center distance of features of the same category in different modalities, the similarity of cross-modality within the category is improved. The loss function of heterogeneous centers is expressed as follows:

(2) $$\begin{array}{c} L_{HC}=\sum_{i=1}^{U}\left[{∥C_{i,1}-C_{i,2}∥}_{2}^{2}\right], \end{array}$$

(3) $$\begin{array}{c} c_{i,1}=\frac{1}{M}\sum_{j=1}^{M}x_{i,1,j}, \end{array}$$

(4) $$\begin{array}{c} c_{i,2}=\frac{1}{N}\sum_{j=1}^{N}x_{i,2,j}, \end{array}$$

where $C_{i,1}$ and $C_{i,2}$ represent the center of the characteristic distribution of the *i*th person in two modalities. Using the same method as [23], to reduce the computational cost, a small batch strategy is used to update the center of feature distribution in two modalities of each batch. It has been verified in [23] that HC loss can only improve the inter-modality similarity within a class, yet cannot enlarge the distance between different categories. Thus, we take the form of supervision training combined with cross-entropy loss and heterogeneous center loss (HC loss). We use the following formula to express the contribution of the two losses:

(5) $$\begin{array}{c} Total\_Loss=CE\_loss+\eta HC\_loss, \end{array}$$

where η is the weight of HC loss in the total loss.

### 3.4. Comparison with HC Loss (Zhu et al., 2020) and AGW (Ye et al., 2021)

Ye et al. [25] applies the idea of non-local attention to the design of the network, taking into account the contribution of the global response of the image to the local response. Although AWG [25] has achieved good performance in single-modality (RGB modality) person re-identification and cross-modality (RGB-IR modality) person re-identification, some improvements are needed to improve the similarity of the intra-class under different modalities. Zhu et al. [23] uses the loss of heterogeneous centers to improve the intra-class similarity of different modalities, but the contribution of the global response of the image to the local positions is not considered in the network designing. Considering the advantages of AWG [25] and HC loss [23], we design a cross-modality pedestrian re-identification network combining heterogeneous center loss and non-local mechanism, which not only solves the problem that local operations cannot see the whole situation clearly but also improves the cross-modality similarity within the class. It provides an excellent baseline for cross-modality person re-identification.

## 4. Experiment

### 4.1. Datasets and Setting

#### 4.1.1. Datasets

The experiments in this paper are carried out on datasets of SYSU-MM01 [26], which is publicly used in cross-modality pedestrian re-identification. SYSU-MM01 [26] is the first published cross-modality pedestrian Re-ID dataset (visible images and infrared images). It contains 491 pedestrians, including 287,628 RGB images (captured with cameras numbered as 1, 2, 4, and 5) and 15,792 IR images (captured with cameras numbered as 3 and 6). SYSU-MM01 [26] contains a training set of 296 pedestrians, a validation set of 99 pedestrians, and a testing set of 96 pedestrians. To make the captured image consistent with the actual situation, the visible light cameras are set in a bright place, and the infrared cameras are set in a dark place. To prevent the influence of different light between indoor and outdoor on the shooting effect, the cameras numbered 1, 2, and 3 are placed indoors, and the cameras numbered 4, 5, and 6 are placed outdoors. The SYSY-MM01 [26] dataset is very challenging due to the changes in camera viewing angle and human postures.

#### 4.1.2. Evaluation Metrics

In the SYSU-MM01 [26] datasets, the training set is used for training. In the testing phase, the query set comes from the IR images, and the gallery set comes from the RGB images. According to the locations of the cameras, the search mode is divided into all-search mode and indoor-search mode. All-search mode contains images from indoors and outdoors. Indoor-search mode contains images from indoors. Single-shot and multi-shot are all included in the all-search mode and indoor-search mode, respectively, and the single-shot all-search mode is used in our experiment. In the evaluation phase, the SYSU-MM01 [26] datasets are repeated multiple times (10 times) during evaluation to take the mean as a relatively stable result. The evaluation indexes that are widely used in person re-identification are CMC, mAP, and Rank-n. CMC is cumulative matching characteristic curve, mAP is mean average precision, and Rank-n is the probability that the first n searched results contain correct samples.

#### 4.1.3. Implementation

The model of GPU used in the experimental environment is an NVIDIA Titan XP, and the experimental code is built under the pytorch framework. To compare with [23] under the same condition, we use the same parameter setting as [23]. The size of the image is set to 288×144. On account of the limited number of pedestrian images in SYSU-MM01 [26], we do some preprocessing operations on pedestrian images to achieve the purpose of data enhancement. Image preprocessing includes random clipping and random horizontal flipping. We use small batch settings of batch size 64. For each batch of the data, 8 RGB images and 8 IR (or thermal imaging) images are selected from each pedestrian image set by a random extraction method. When the feature map is evenly partitioned horizontally, the number of blocks is set to 6. We use a convolution kernel with a size of 1 to reduce the dimension of the eigenvector (512). When calculating loss, the weights of cross-entropy and HC loss are set to 1 and 0.5, respectively. The value of epoch is set to 60. Using the warmup dynamic learning rate adjustment strategy, the learning rate is initialized to 0.01. When the epochs are between 30 and 60, we change the learning rate to 0.001. The optimization algorithm adopts the stochastic gradient descent with momentum 0.9.

### 4.2. Comparison with Mainstream Methods

In our comparative experiments, we enumerate several non-deep feature learning methods (LOMO [8], HOG [45]) and several deep learning methods in cross-modality person Re-ID and compare them with this paper. The methods of deep learning are zero-padding [26], TONE+HCML [46], BDTR [20], and HC-loss [23], as shown in Table 1. The baseline of this paper is inspired by HC-loss [23]. As shown in the data in Table 1, the accuracy of rank-1 has been steadily improved after adding non-local to the feature extraction module. We also apply the PCB method, which was originally used for person Re-ID in visible light modality, to cross-modality person Re-ID. The experimental results show that if we apply the single-modality method to cross-modality person Re-ID without changing anything, the effect will be significantly reduced. The reason is that the differences between different modalities are not considered.

### 4.3. Ablation Study

We compare the performance between without using HC loss and with using HC loss, as shown in Table 2. The first row in Table 2 shows the effect of our experiment when combining the use of HC loss and CE loss. The second row in Table 2 shows the recognition accuracy of our experiment using CE loss alone. The data in Table 2 shows that if CE loss is used alone, the recognition accuracy will be reduced. When HC loss and CE loss are used together, the recognition accuracy is improved. The reasons are as follows: firstly, the CE loss is used to widen the characteristic distance of different types of pedestrians in the general direction, and then HC loss is used to reduce the center distance between different modalities in the class. CE loss and HC loss can be jointly beneficial to re-identification. The data used for verification are shown in Table 2.

We compare the performance between without using the non-local module and with using the non-local module, as shown in Table 3. If we add non-local to the shallow input with a large size, the computation will be very heavy. So we choose to add the non-local module in the high-level semantic rich layer. The two integer-type parameters in parentheses indicate where the non-local module is embedded. The third row in Table 3 shows that when we choose to add the non-local in the second and third layers, the performance of recognition is the best. The effect of adding non-local to other layers is shown in Table 3.

## 5. Conclusions

In this work, we propose a dual-channel deep network combining heterogeneous center loss and non-local features. To improve the contribution of global response to local response, a non-local module is embedded in the high-order semantic layer of the network. Before average pooling, we use the method of horizontal segmentation to extract the features of each part of the human body. Finally, we concatenate the features of each horizontal segmentation into a whole for cross-modality person Re-ID. When calculating the loss function, we use cross-entropy loss and heterogeneous center loss to enlarge the characteristic distance between different pedestrians and reduce the characteristic distance of the same pedestrians in different modalities. Experimental results show that our method achieves good performance. Our method can provide a good baseline for subsequent cross-modality person Re-ID. In the next stage, we will attempt using this method to study multi-modality re-identification.

## Figures and Tables

**Table 1:** Comparison of experimental results in single-shot all-search mode on SYSU-MM01 [26] datasets. The data unit is %.

Method	Rank-1	Rank-10	Rank-20	mAP
HoG+Euclidean [45]	2.76	18.25	31.91	4.24
HoG+KISSME [45]	2.12	16.21	29.13	3.53
HoG+LFDA [45]	2.33	18.58	33.38	4.35
LOMO+CCA [8]	2.42	18.22	32.45	4.19
LOMO+CDFE [8]	3.64	23.18	37.28	4.53
LOMO+GMA [8]	1.04	10.45	20.81	2.54
Asymmetric FC [26]	9.30	43.26	60.38	10.82
One-stream [26]	12.04	49.68	66.74	13.67
Two-stream [26]	11.65	47.99	65.50	12.85
Zero-padding [26]	14.80	54.12	71.33	15.95
PCB [11]	16.43	54.06	65.24	16.26
TONE+HCML [46]	14.32	53.16	69.17	16.16
BDTR(AlexNet) [20]	20.84	63.81	79.14	22.86
BDTR(ResNet50) [20]	27.32	66.96	81.07	27.32
eBDTR(ResNet50) [20]	27.82	67.34	81.34	28.42
cmGAN(ResNet50) [42]	26.97	67.51	80.56	27.80
HC-loss [23]	55.96	90.51	96.19	55.07
Ours	58.09	93.14	97.42	58.30

**Table 2:** Our experiment with HC loss and without HC loss. The data unit is %.

Method	Rank-1	Rank-10	Rank-20	mAP
Ours	58.09	93.14	97.42	58.30
Ours without HC	46.78	86.13	93.18	46.13

**Table 3:** Our experiment with non-local module and without non-local module. The data unit is %.

Method	Rank-1	Rank-10	Rank-20	mAP
Ours without Non-local	55.96	90.51	96.19	55.07
Ours Non-local (1, 2)	55.56	93.16	97.34	55.82
Ours Non-local (2, 3)	58.09	93.14	97.42	58.30
Ours Non-local (3, 4)	57.30	92.95	97.37	56.56

## Data Availability

The dataset source are listed in the paper.
